# The Use of Contrast-Enhanced Post Mortem CT in the Detection of Cardiovascular Deaths

**DOI:** 10.1371/journal.pone.0093101

**Published:** 2014-04-23

**Authors:** Jonas Christoph Apitzsch, Saskia Westphal, Tobias Penzkofer, Christiane Katharina Kuhl, Ruth Knüchel, Andreas H. Mahnken

**Affiliations:** 1 Department of Diagnostic and Interventional Radiology, Philipps University Hospital Marburg, Marburg, Germany; 2 Department of Pathology, RWTH Aachen University Hospital, Aachen, Germany; 3 Applied Medical Engineering, Helmholtz Institute, RWTH Aachen University, Aachen, Germany; 4 Department of Diagnostic and Interventional Radiology, RWTH-Aachen University Hospital, Aachen, Germany; Centre Hospitalier Universitaire Vaudois, switzerland

## Abstract

**Objectives:**

To evaluate the diagnostic value of contrast enhanced post mortem computed tomography (PMCT) in comparison to non-enhanced post mortem CT in the detection of cardiovascular causes of death (COD).

**Background:**

As autopsy rates decline, new methods to determine CODs are necessary. So contrast enhanced PMCT shall be evaluated in comparison to established non-enhanced PMCT in order to further improve the method.

**Methods:**

In a prospective study, 20 corpses were examined using a 64-row multisclice CT (MSCT) before and after intraarterial perfusion with a newly developed, barium-bearing contrast agent and ventilation of the lungs. The cause of death was determined in enhanced and unenhanced scans and a level of confidence (LOC) was given by three experienced radiologists on a scale between 0 and 4. Results were compared to autopsy results as gold standard. Autopsy was performed blinded to PMCT-findings.

**Results:**

The method allowed visualization of different types of cause of death. There was a significant improvement in LOC in enhanced scans compared to unenhanced scans as well as an improvement in the detection of COD. The cause of death could be determined in 19 out of 20 patients.

**Conclusions:**

PMCT is feasible and appears to be robust for diagnosing cardiovascular causes of death. When compared with unenhanced post-mortem CT intraarterial perfusion and pulmonary ventilation significantly improve visualization and diagnostic accuracy. These promising results warrant further studies.

## Introduction

Death due to cardiac disease is the leading cause of death worldwide [1]. 864000 Patients die each year due to cardiac death in the United States alone [2]. Normally, these diseases are found in conventional autopsy, but autopsy rates have been declining in Germany and all over the world over the last years [3]–[9]. Therefore, it is desirable that alternative methods such as PMCT be further improved in order to detect cardiovascular diseases.

In a recent study, our group has shown, that PMCT is a very valuable tool in the diagnosis of the cause of death, complementing conventional autopsy in many ways, but that conventional autopsy still is the unsurpassed gold standard [10].

To reduce the gap in diagnostic accuracy between conventional autopsy and PMCT, we assessed the value of contrast enhanced PMCT in the diagnosis of cardiovascular related diseases.

PMCT has become more and more common over recent years [11], [12].

It is an established method in forensic medicine to find the cause of death [13], [14]. Different approaches have been made in order to increase the diagnostic value of PMCT. One of these approaches has been the effort to inject different kinds of contrast agents into the vascular system in order to achieve a contrast enhanced scan [11], [15], [16]. Different methods and contrast agents have been tested in order to achieve a satisfying contrast as well as different kinetics of the applied agent. Limitations of these studies have always been leaking of contrast medium into extravasal space with oily solutions or adherent edema with water soluble solutions [16]. Further, reduced diagnostic accuracy due to post-mortem lung changes have been published [15].

In this study a new method was evaluated in order to achieve a good contrast enhancement of the complete arterial system and to compare the diagnostic accuracy in the detection of cardiovascular deaths to that of unenhanced PMCT and conventional autopsy.

## Methods

In a prospective study, 20 human bodies (13 male, 7 female, 28 to 85 years old, median age 68,05 years) have been examined using multislice computed tomography (MSCT) before and after intraarterial contrast enhancement and pulmonary ventilation. Ethical approval was given by the RWTH-Aachen University Hospital Ethics Committee (EK 051/11), and all relatives gave written informed consent for each procedure.

All scans were acquired within the first 24 hours after death in order to avoid decomposition. Each CT scan was evaluated by three different, experienced radiologists independently and without knowledge of clinical history or circumstances of death in order to avoid bias. All Radiologists had substantial experience in reporting CT, neuroradiology and cardiac CT imaging. All examined corpses thereafter underwent conventional autopsy. The performing pathologists had no knowledge of PMCT-findings. Enhanced and unenhanced CT findings were compared with each other and thereafter to autopsy findings that were used as reference standard. All findings can be seen in Table 1.

**Table 1:** 

Sex	COD in unenhanced PMCT	COD in contrast enhanced PMCT	COD in conventional autopsy
m	Enlarged heart - cardiac failure	occluded venous bypasses and enlarged heart - cardiac failure due to MI	Cardiac failure, occluded venous bypasses, myocardial infarction
m	pulmonary metastasized tumour burden	pulmonary metastasized tumour burden	Multiorgan failure, pulmonary metastasized tumour burden
f	Heamatopericard leading to tamponade	Heamatopericard leading to tamponade	Heamatopericard and right ventricular failure
m	Tumour assumed cause of death	Tumour assumed cause of death	Tumour associated multi organ failure
f	Cardiac failure with cardiac hypertrophy and pulmonary edema	Cardiac failure with cardiac hypertrophy and pulmonary edema	Myocardial infarction, pulmonary edema and consecutive heart failure
m	Mesenteric infarction	Mesenteric infarction	Aortic endocarditis and mesenteric ischemia
m	Aortic dissection, haematopericard, pericard rupture and large haematoma in the pleural space with consecutive exsanguination	Aortic dissection, haematopericard, pericard rupture and large haematoma in the pleural space with consecutive exsanguination	Aortic dissection Stanford A and haemorrhagic shock
m	Cardiac failure assumed cause of death due to enlarged heart and pleural effusion	Cardiac failure assumed cause of death due to enlarged heart and pleural effusion	Cardiac decompensation due to cardiomyopathy and advanced atherosclerosis
f	No cause of death found	No cause of death found	Toxic Shock Syndrome
m	Hemorrhagic shock due to exsanguination	Hemorrhagic shock due to suture insufficiency of the ascending aorta and exsanguination	Hemorrhagic shock due to suture insufficiency of the ascending aorta
f	Cardiac failure assumed cause of death due to enlarged heart, postoperative situs and pulmonary edema	Cardiac failure assumed cause of death due to enlarged heart, postoperative situs and pulmonary edema	Rejection of transplanted heart with myocardial infarction
f	Respiratory insufficiency with ground-glass like lung	Respiratory insufficiency with ground-glass like lung	Cardiorespiratory insufficiency with hyaline membranes
m	Suspected cardiac failure due to enlarged heart, pulmonary edema and ascites	Suspected cardiac failure due to enlarged heart, pulmonary edema and ascites	Biliary peritonitis after suture insufficiency in Billroth II. Heart failure due to elevated preload
m	pulmonary embolism and Cardiac failure. Embolus in IVC	pulmonary embolism and Cardiac failure. Embolus in IVC	Cardiac failure and pulmonary embolism. Embolus in IVC.
m	Right ventricular rupture and haemorrhagic shock	Right ventricular rupture and haemorrhagic shock	Right ventricular rupture and haemorrhagic shock
f	Rupture of the intraventricular septum and thrombosis of the aorta and aortic valve	Rupture of the intraventricular septum and thrombosis of the aorta and aortic valve with obstruction of left coronary artery	Myocardial infarction with rupture of the intraventricular septum and thrombosis of the aorta
m	Infarction of almost the entire right hemisphere	Embolus in the right internal carotid artery and Infarction of almost the entire right hemisphere	No cause of death could be determined (no consent from family to examine brain through autopsy)
m	Cardiac insufficiency	acute Cardiac failure	acute cardiac failure
m	right ventricular failure	acute right ventricular failure	right ventricular failure
m	Hemmorrhage and shock	Hemmorrhage and shock due to aortic rupture	Hemmorrhage and shock due to aortic rupture

### Preparation, Ventilation, Contrast Administration and Scan Settings

The unenhanced scans were acquired without the use of contrast agent. The scan parameters were the same as with the enhanced scans and can be seen further on in the text.

**Figure 1:**
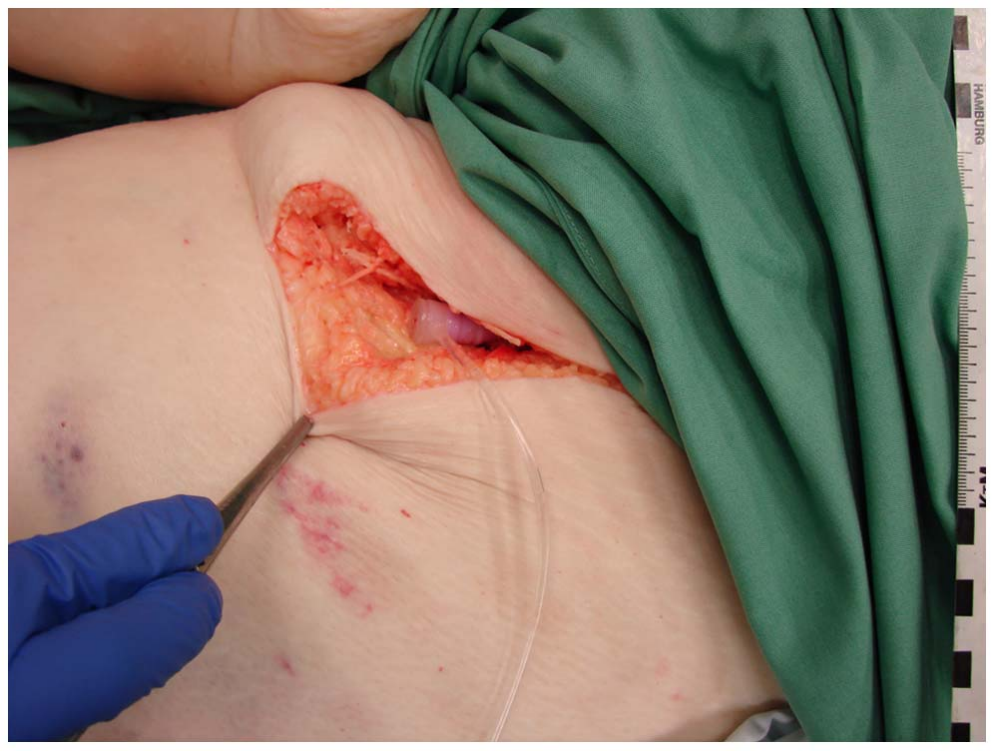
Sheath in the left common femoral artery after surgical preparation.

Prior to the enhanced scan, both femoral arteries were surgically exposed and cannuled with an 18 Gauge needle (Abbocath). A 0.89 mm Terumo guidewire was inserted over the needle to catheterize the vessel. Thereafter, a 10 French Sheaths was inserted into each of the vessels over the wire (Figure 1). The person who's hands can be seen on the image has given written informed consent, as outlined in the PLOS consent form, to publication of their photograph.

A viscous solution of water and cellulose was mixed with barium-bearing contrast medium (Micropaque).

Oily liquids often result in a good visualization of even very small vessels [17]. But they often penetrate the surrounding tissue and can cause perivascular edema [18]. Water soluble contrast agents often extravasate very quickly into the interstitial space [17], [19] and most often lack the ability to flush postmortem clots [20], [21].

The latter has been proved to be due to the lesser viscosity of water soluble contrast agents [22].

Thus, we developed a new contrast agent that would neither extravasate nor cause perivascular edema and still penetrate smallest vessels. Therefore, for intraarterial contrast enhancement, we mixed a solution that was both viscous and water soluble. In order to provide viscosity, we mixed a cellulose and water solutionwith Mikropaque. The solution was titrated to a density of 300 Hounsfield Units (HU) using the same scan parameters as in the PMCTs. Directly before perfusion, the solution was well mixed in order to avoid sedimentation. Contrast homogeneity of the solution was tested scanning a large amount of solution prior to intravascular administration.

**Figure 2:**
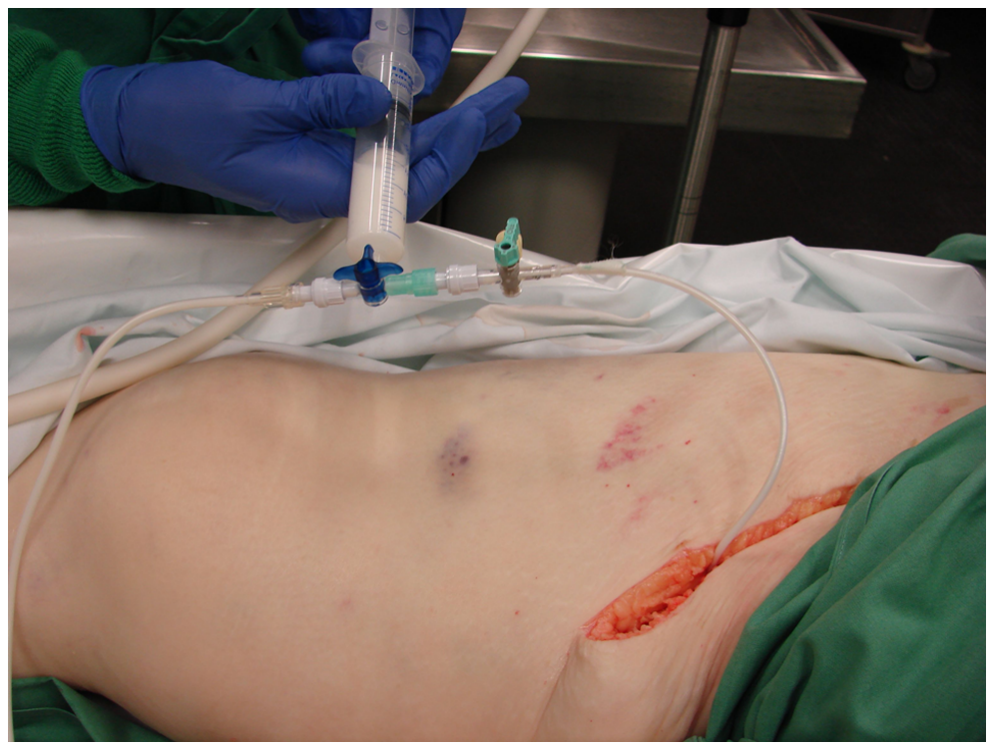
Manual perfusion with a 20 ml syringe via the right common femoral artery.

**Figure 3:**
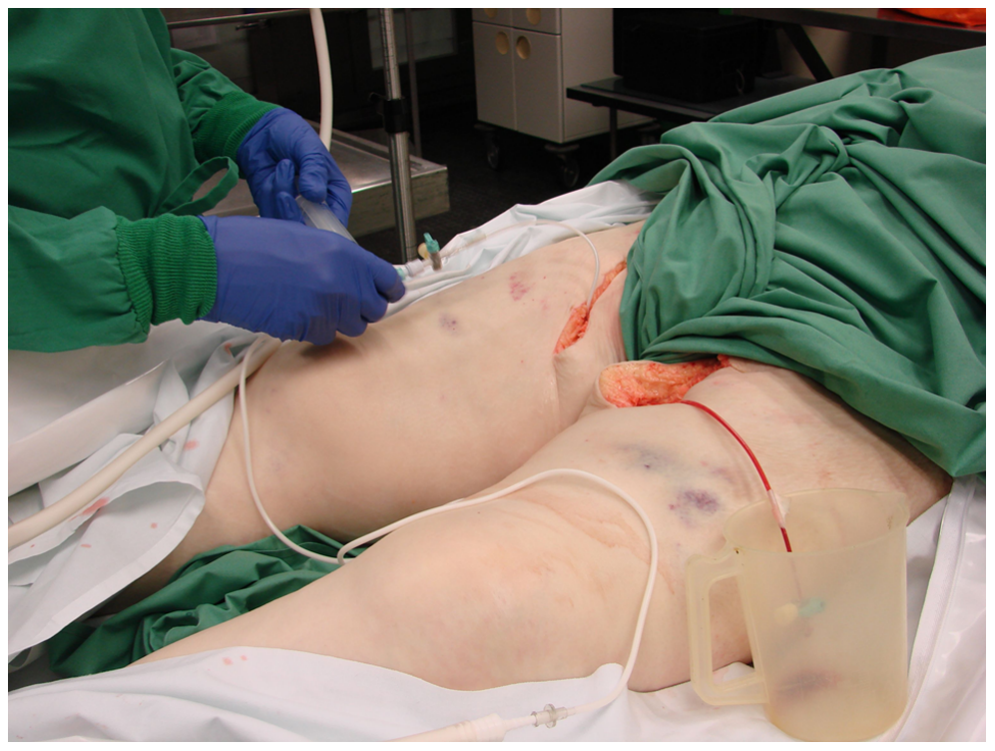
Exiting of blood and contrast medium over the sheath in the left common femoral artery.

The contrast medium was manually administered via the sheath in the right common femoral artery with a 20 ml syringe (Figure 2). The person who's hands can be seen on the image has given written informed consent, as outlined in the PLOS consent form, to publication of their photograph. When blood was aspirable, the arterial perfusion with the solution was started until it exited via the contralateral sheath (Figure 3). In most cases, less than 2 l of contrast solution were necessary.

The lungs were ventilated with a laryngeal mask (AERO TUBE Laryngeal Mask, size 3) that was sealed with a clamp at the moment of maximum filling of the lungs, thus resulting in a “maximum inspiration” state at the time of the scan in order to distend the thorax. Secure placement of the mask was reached by auscultation of the chest.

To avoid beam hardening artefacts, the arms were elevated over head level. Scan parameters were as follows:

CT was performed using a SOMATOM Definition CT system (Siemens Medical Solutions, Forchheim, Germany). A tube setting of 120 kV with variable mAs was applied. A collimation of 5.0, 1.0 and 0.75 mm, a pitch of 0.65 and a rotation time of 0.33 s were used. The entire body was scanned in a single scan. Further, cranium, thorax abdomen and heart were scanned separately.

The raw data was reconstructed with soft tissue, lung tissue and bone tissue algorithm in Kernel B20, B50 and B70. 3D data and maximum intensity projections were developed as well.

All corpses underwent unenhanced scan prior and directly after contrast administration in “maximum inspiration”-state.

All scans were thereafter evaluated independently by the three above mentioned radiologists. All had to give a full summary of their findings, an interpretation and a cause of death. If the cause of death could not be definitely determined, each radiologist had to give their opinion on the most likely cause of death.

Criteria for analysis included enhancement of vessels, arterial stenosis or occlusion, myocardial thickness, lung ventilation, extravasation of contrast medium and gross pathologies such as tumours or big haematomas.

Vessel stenosis of up to 50% was regarded as non-significant, 50%–75% as significant and more than 75% as severe stenosis.^1^

### Statistical Methods

All data was analyzed using SPSS version 21 and Microsoft Excel version 14.0.

Mean LOC was calculated for each patient. An error bar was calculated and can be seen below. Demographic data was measured using Microsoft Excel. The Wilcoxon Test for non-parametric tests was used to compare LOC in both groups.

## Results

**Figure 4:**
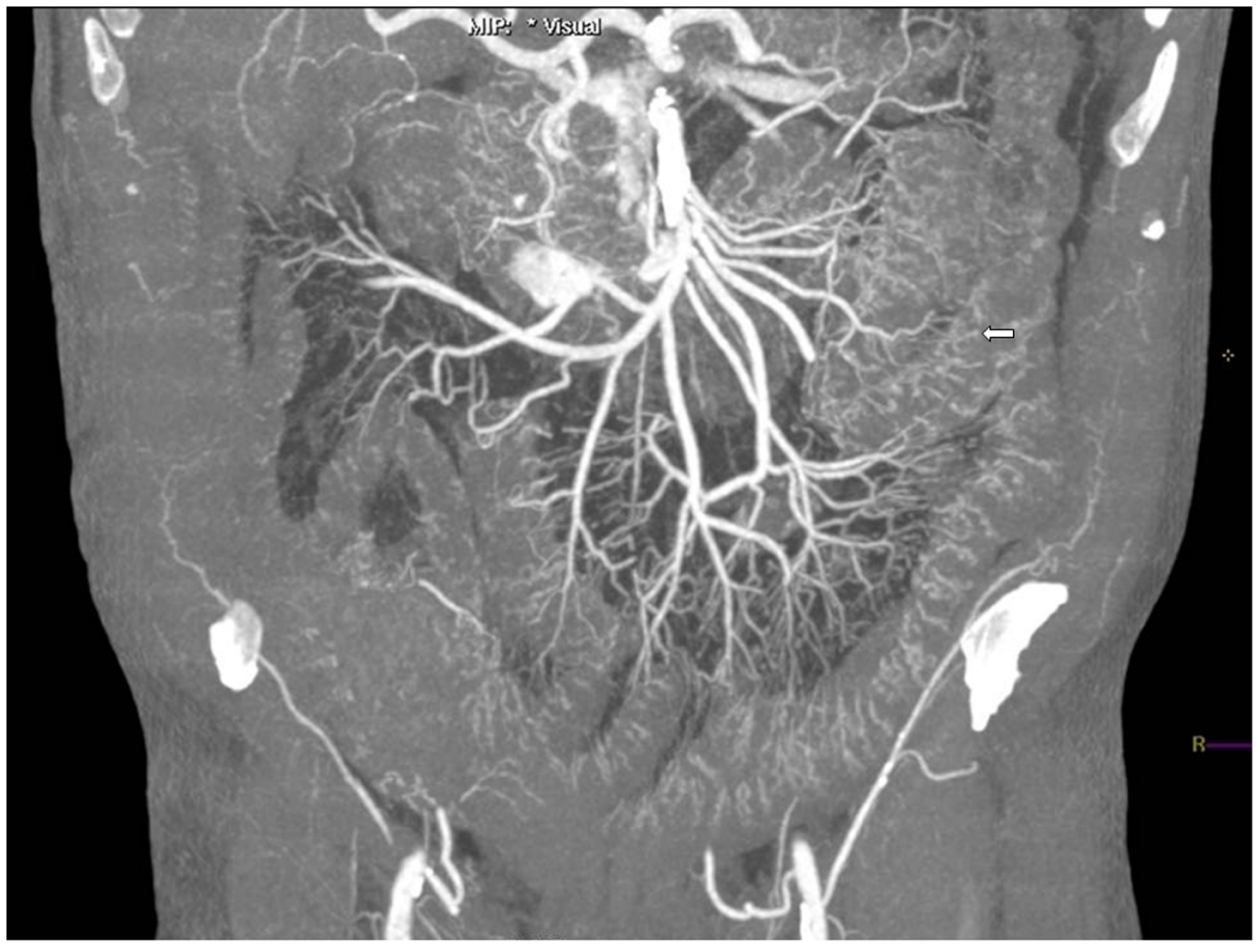
Maximum intensity projection of the lower abdomen. The contrast medium can be very well seen in even small visceral arteries and arteries of the jejunal wall (white arrow).

The authors experienced no difficulties preparing the patients. Preparation and catheterization of the vessels as well as intraarterial perfusion were easily manageable in each of the patients. Placement of the laryngeal mask and ventilation of the lungs went smoothly each time. Postmortem perfusion resulted in a very good arterial vessel opacification with homogenous contrast enhancement of even very small vessels (Figure 4). Contrast medium could be followed down to small vessels of a size of 0.75 mm. The newly developed contrast agent showed a very homogeneous contrast with equally measurable 300 Hounsfield Units in the left ventricle, descending aorta, common carotid arteries, common iliac arteries and popliteal arteries. There were no cases in which the coronary vessels or coronary bypasses were not enhanced due to methodical failures. No extravasation or shunting to the venous system occurred. Even after full perfusion of the arterial system and contrast medium exiting via the contralateral sheath did the solution not enhance the veins or extravasate. All Radiologists had to give a diagnosis with Cause Of Death (COD) and a Level Of Confidence (LOC) in their determination of the cause of death for unenhanced and contrast-enhanced scans [23]. Level of Confidence could vary between unascertained (1), possible (2), probable (3) and definite (4). The enhanced scans proved to provide radiologists with a significantly higher LOC in their determination of the cause of death (p: 0,001) than unenhanced scans. Mean LOC for unenhanced scans was 1,65, mean LOC for contrast-enhanced scans was 3,12. The Increase of certainty for the correct diagnoses (as proven through conventional autopsy) for enhanced over unenhanced PMCT was 86%.

Different cardiovascular CODs could be seen during this study.

**Figure 5:**
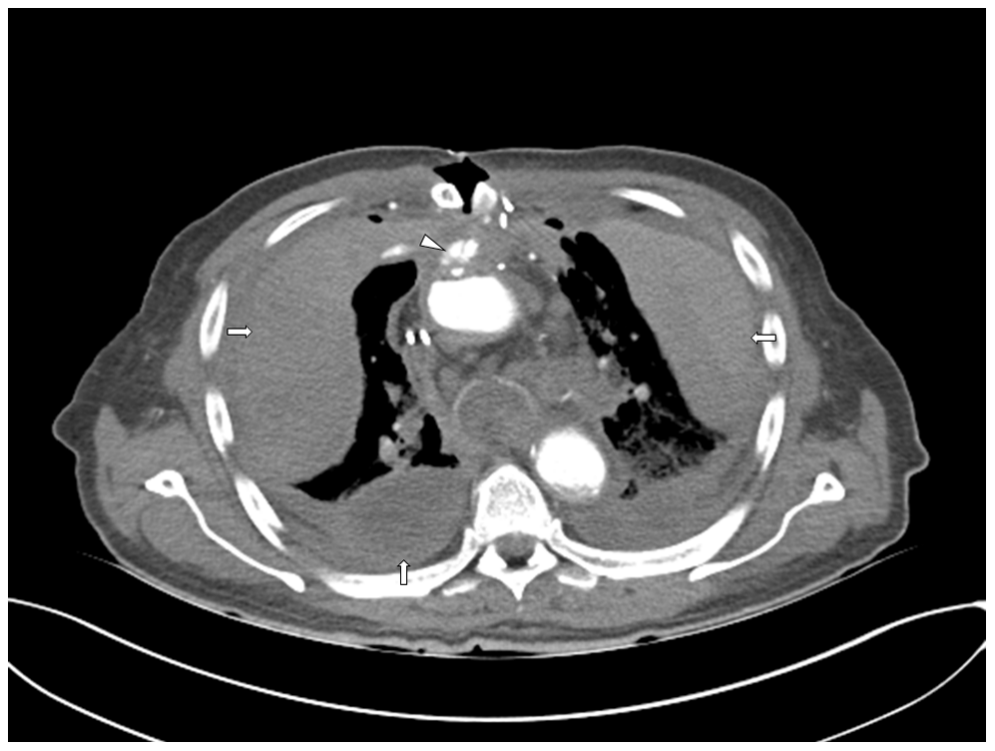
Axial view of the chest, soft tissue-windowing. Big hematomas in the pleural space can be seen (white arrows). Further, the leak of contrast medium from the aortic root directly posterior to the sternotomy can be very well depicted (white arrowhead). Autopsy later confirmed a suture insufficiency and bleeding of aortic root after surgical valve operation.

**Figure 6:**
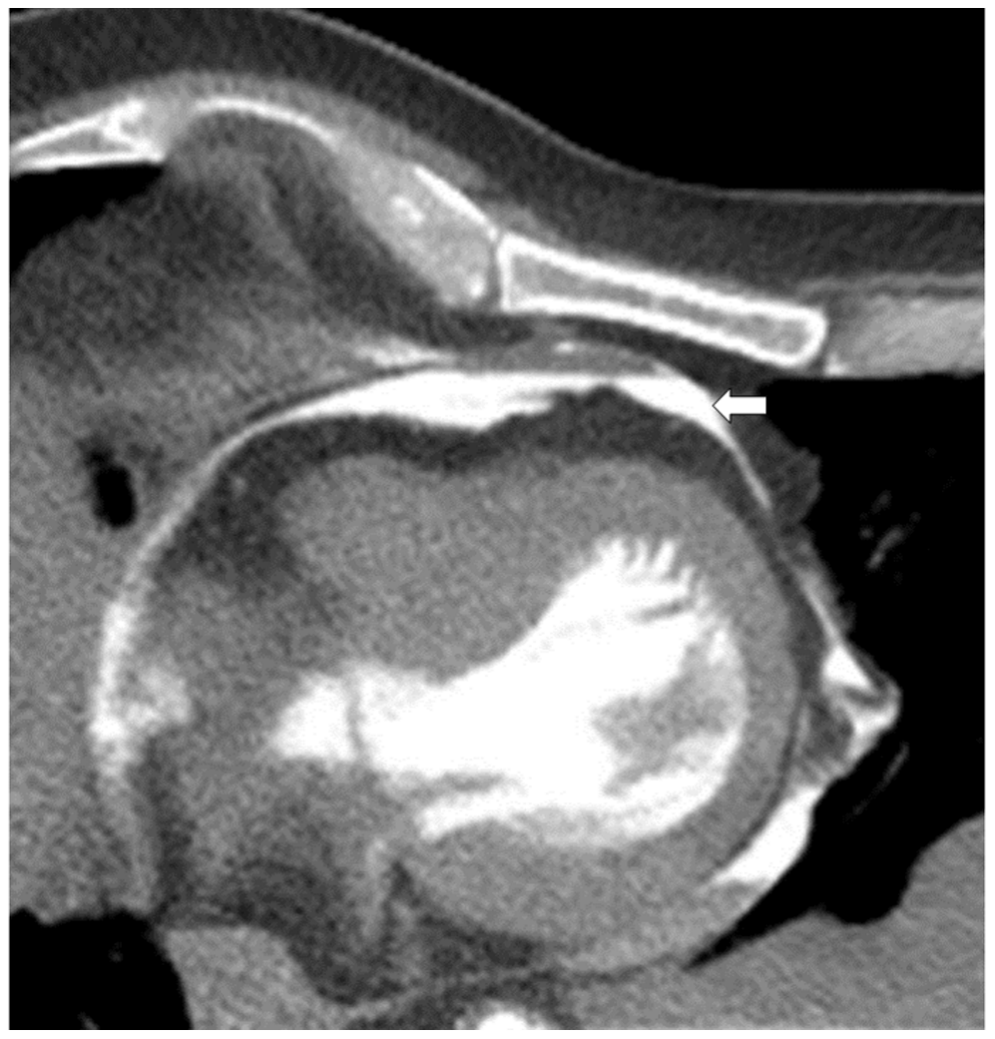
Axial view of the heart, soft tissue-windowing. The scan shows contrast medium between heart and pericardium (white arrow). Autopsy later confirmed an aortic dissection with bleeding into the pericardial space, pericardial rupture and bleeding into the left pleural space which can be seen in Figures 8, 9 and 10.

**Figure 7:**
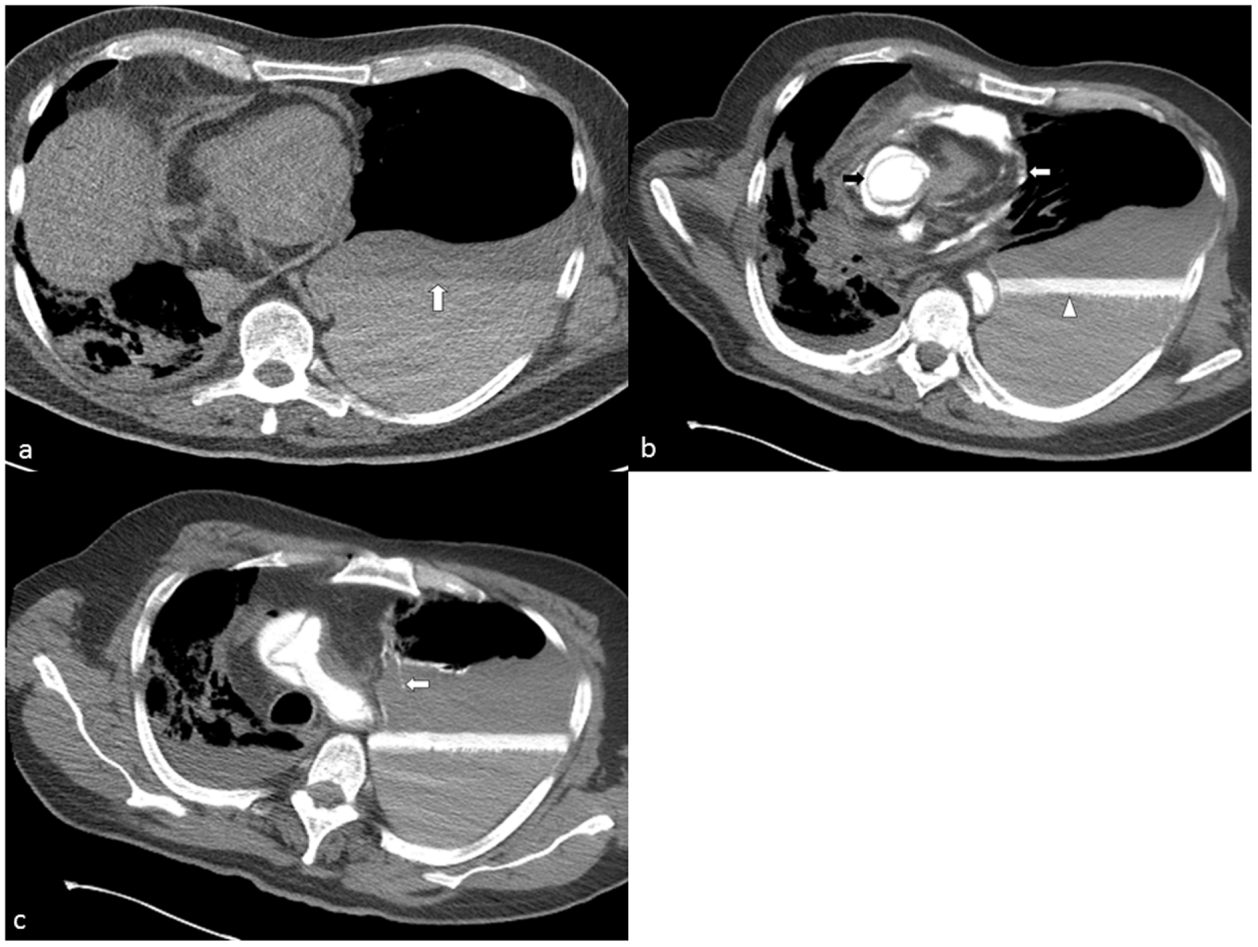
a: Axial view of the chest, soft tissue-windowing. The unenhanced scan shows a big pleural effusion on the left with sedimentation phenomenon (white arrow). The medistinum is pushed to the right with consecutive dystelectasis of the right lung. b: Axial view of the chest, soft tissue-windowing. The contrast enhanced scan of the same patient very well depicts the aortic dissection (black arrow), the pericardial effusion (white arrow) and the blood and contrast medium in the pleural space (white arrowhead). The contrast medium turned out to be heavier than plasma and lighter than blood cells leading to a clear delineation between the two in the hematoma. All findings were later confirmed by autopsy. c: Axial view of the chest, soft tissue-windowing. The Contrast medium leaks downwards from the rupture in the pericardium into the pleural space (white arrow).

**Figure 8:**
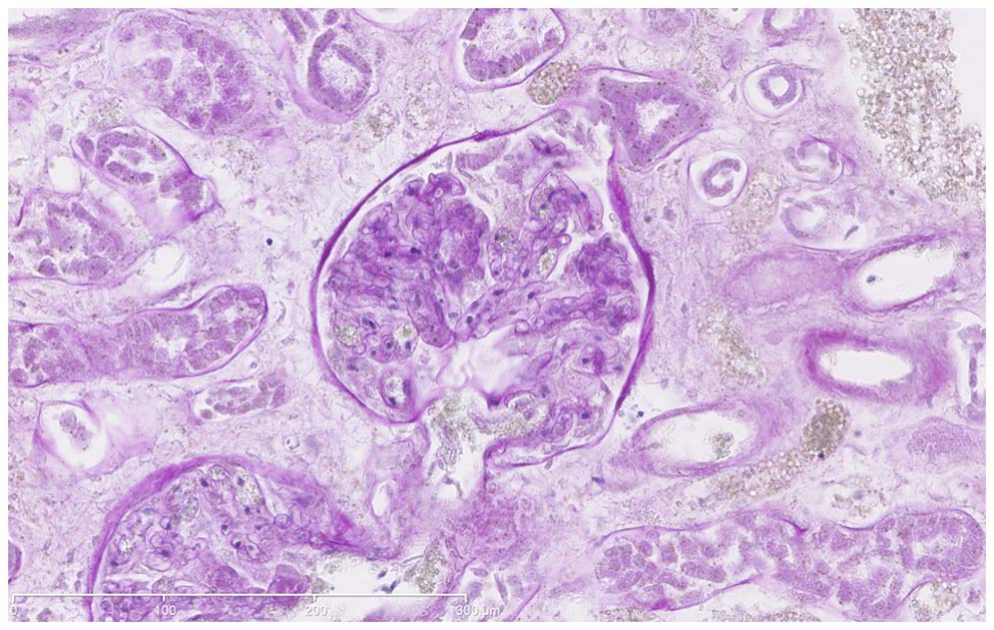
Histologic sample of a renal glomerulum with afferent arteriole (black arrow) and glomerular capillaries (white arrow) filled with contrast medium (PAS, 100x). Thus, histology proved the perfusion of smalles arteries without unintentional extravasation or shunting to the venous system.

**Figure 9:**
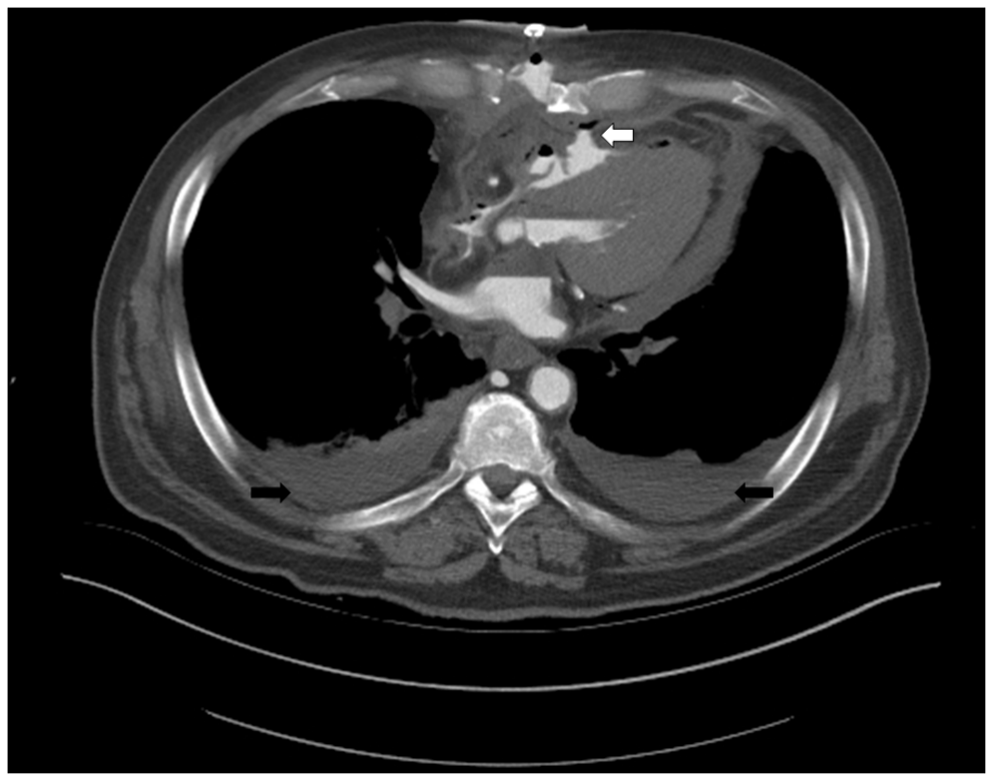
Axial view of the chest, soft tissue-windowing. Rupture of the right ventricle (white arrow) with contrast medium exiting into the sternal cavity and dorsal heamatomas in the pleural space (black arrows).

**Figure 10:**
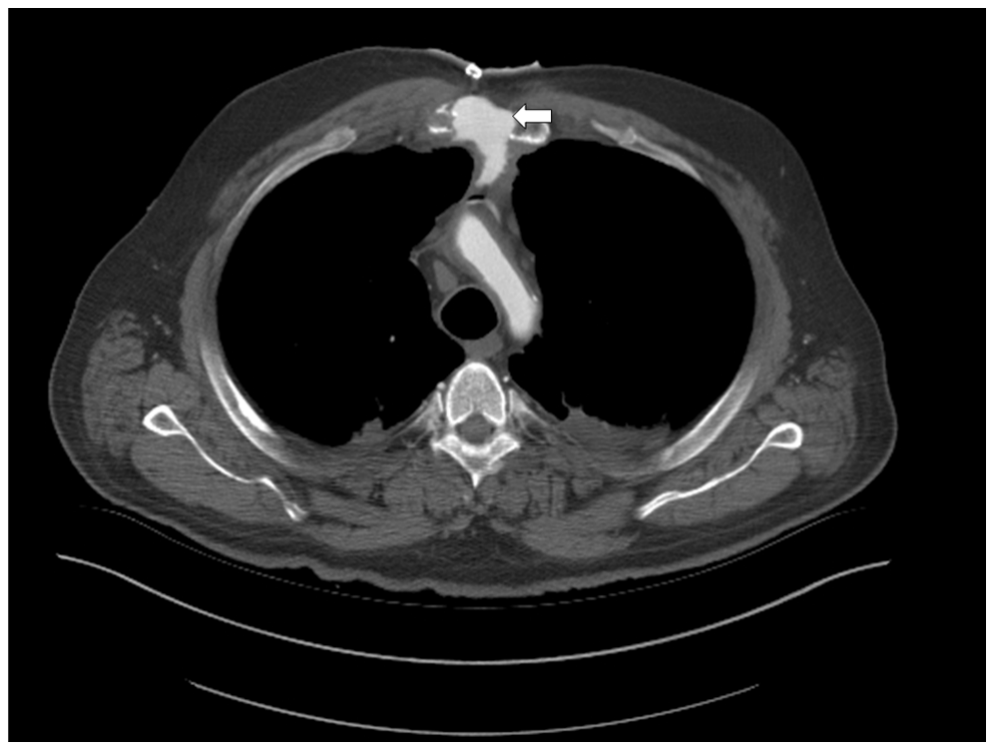
Axial view of the chest, soft tissue-windowing. The exiting contrast medium can very well be seen in the dehiscent sternum, directly posterior to the sternal skin clips (white arrow).

**Figure 11:**
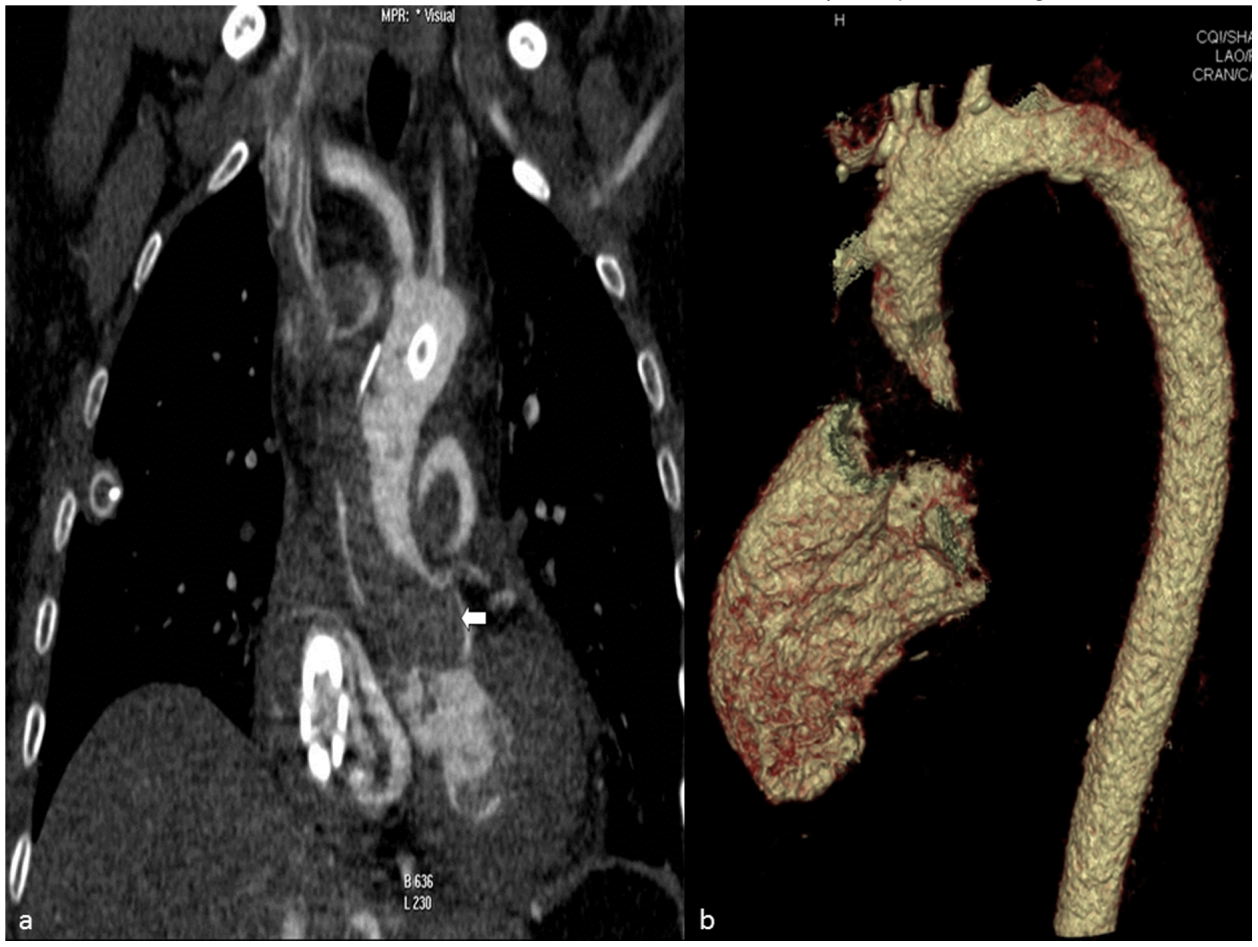
a: paracoronal view of the chest, soft tissue-windowing. Thrombosis of the ascending aorta with occlusion of coronary arteries can be seen (white arrow). Conventional autopsy later confirmed the diagnosis. b: 3D reconstruction of CT of the same patient shows lack of contrast between left ventricle and ascending aorta due to thrombosis.

The method allowed excellent visibility of bleedings (Figure 5), pericardial Effusion due to intrapericardial bleeding (Figure 6) aortic dissection (Figure 7b,c) and insufficiency of surgical sutures. The contrast medium turned out to be lighter than clotted blood and heavier than plasma, leading to a sedimentation phenomenon in older heamatomas (Figure 7a,b,c before and after contrast enhancement). But the contrast medium did indeed reach smallest arterial vessels such as the glomeruli and the vas afferens of the kidney, which was later histologically proven during conventional autopsy (Figure 8). Further, a case of ventricular rupture, sternal dehiscence and bleeding into the opened sternum could very well be depicted due to contrast enhancement (Figures 9, 10). The diagnosis of thrombosis of the aortic valve with occlusion of coronary arteries and rupture of the intraventricular septum could only be made with contrast enhanced PMCT (Figures 11a/b, 12). The ventilation of the lungs led to the diagnosisof antemortem ground glass opacification due to pulmonary edema (Figure 13a,b). In Figure 14, a bleeding due to an insufficiency of a surgical suture can be seen (Figure 14). One patient developed unisocoria during cardiac surgery and died shortly afterwards. PMCT proved an occlusion of the right internal carotid artery with consecutive Infarction of the right hemisphere (Figure 15). As there were several cases where clotted blood in a vessel potentially led to the death of the patient, the question arose, whether the above mentioned clots formed before or after the time of death, especially with the 24 hour time window between death and PMCT. This could until now not be answered with PMCT alone. However, the absence of clots in other scans as well as in other localizations in the same scans led to the assumption that these clots probably formed before deaths. And histopathologic analysis of these clots allowed the differentiation between antemortem clots and post-mortem cruor, thus verifying the diagnoses each time.

**Figure 12:**
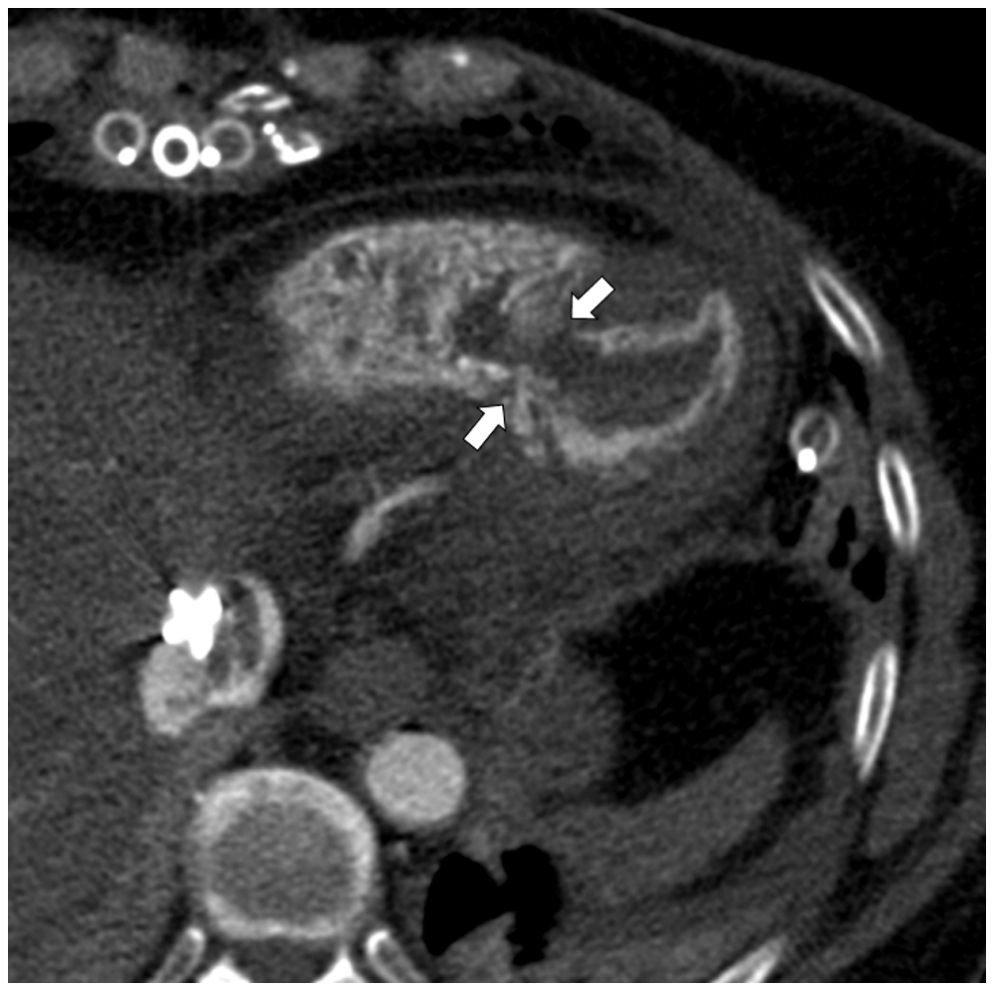
Axial view of the heart, soft tissue-windowing. The large rupture of the intraventricular septum with contrast medium entering the right ventricle can very well be seen.

**Figure 13:**
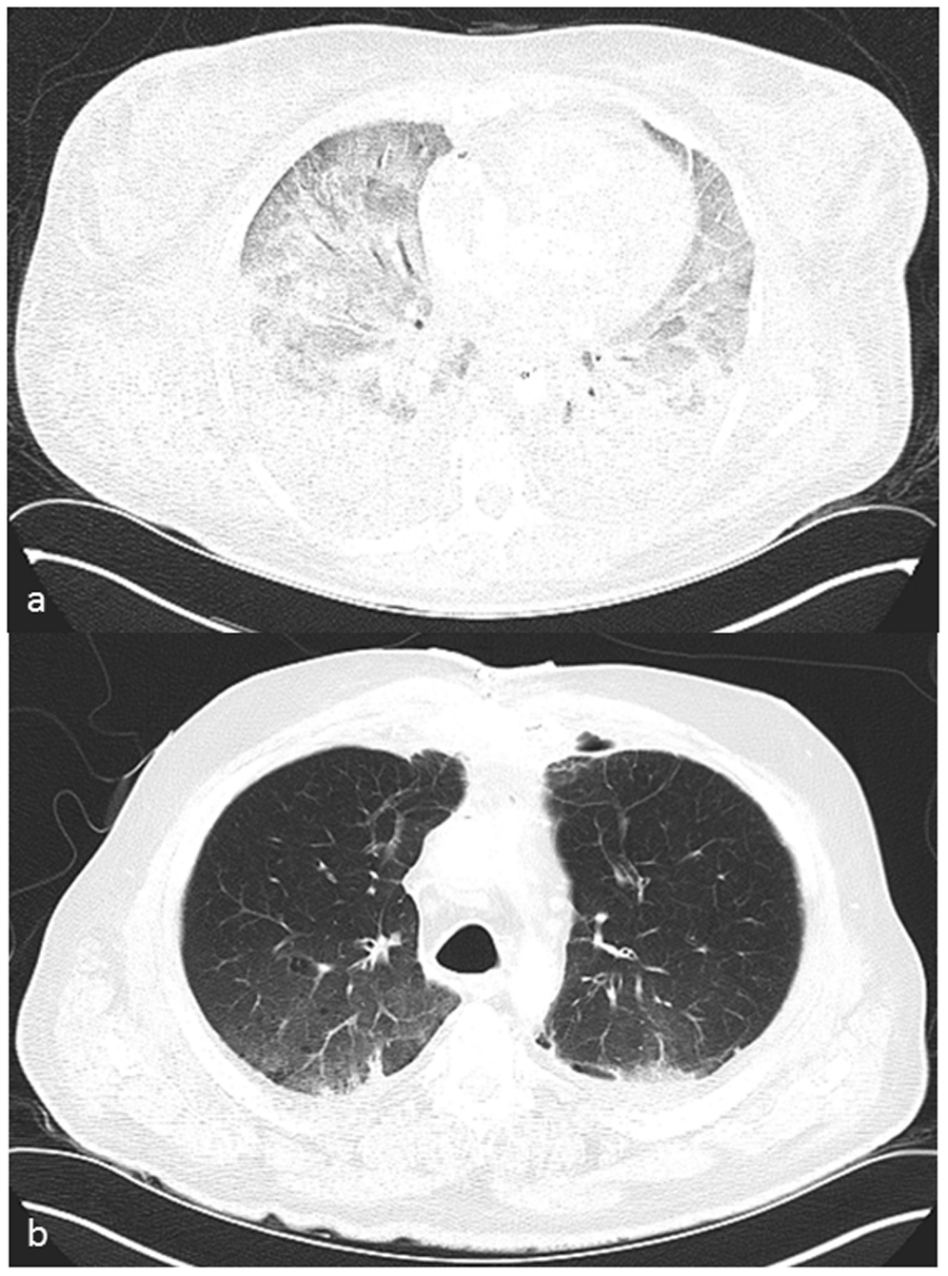
a: axial view of the chest, lung windowing. Ground-glass like lung and pleural effusion due to pulmonary edema. Yet no collapse of the lungs because of postmortem ventilation. b: axial view of the chest, lung windowing. Normally expanded lungs because of postmortem ventilation.

**Figure 14:**
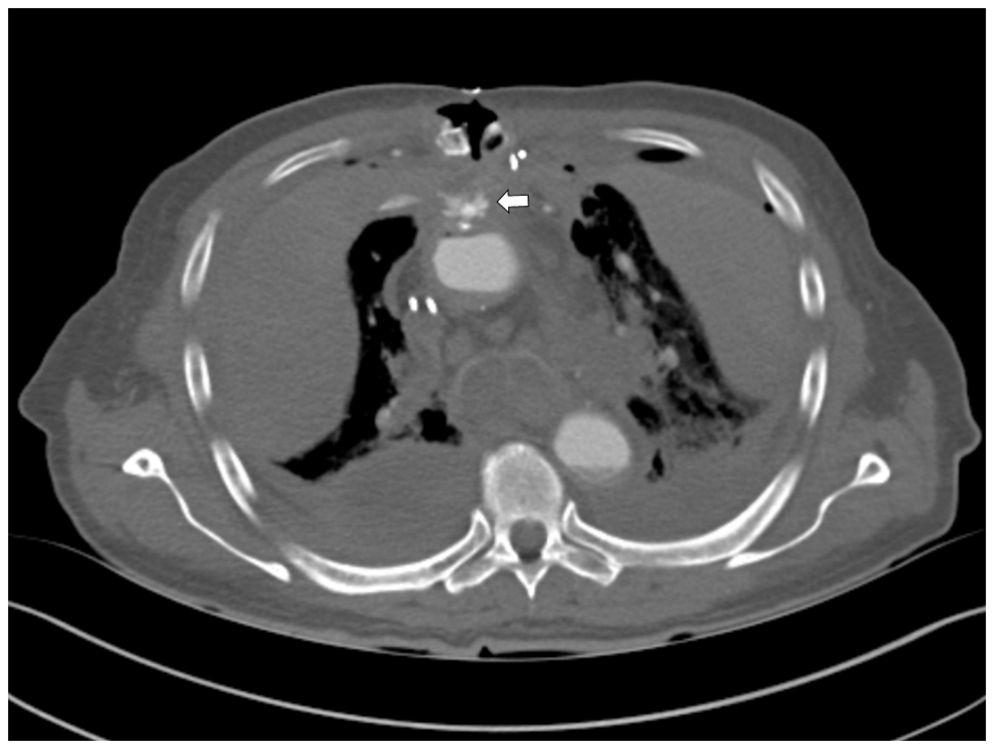
Axial view of the chest, mediastinal windowing. Haematomas in the thorax. The exiting of contrast medium from the aortic root can be very well depicted (white arrow). Autposy later confirmed suture insufficiency of the aortic root.

**Figure 15:**
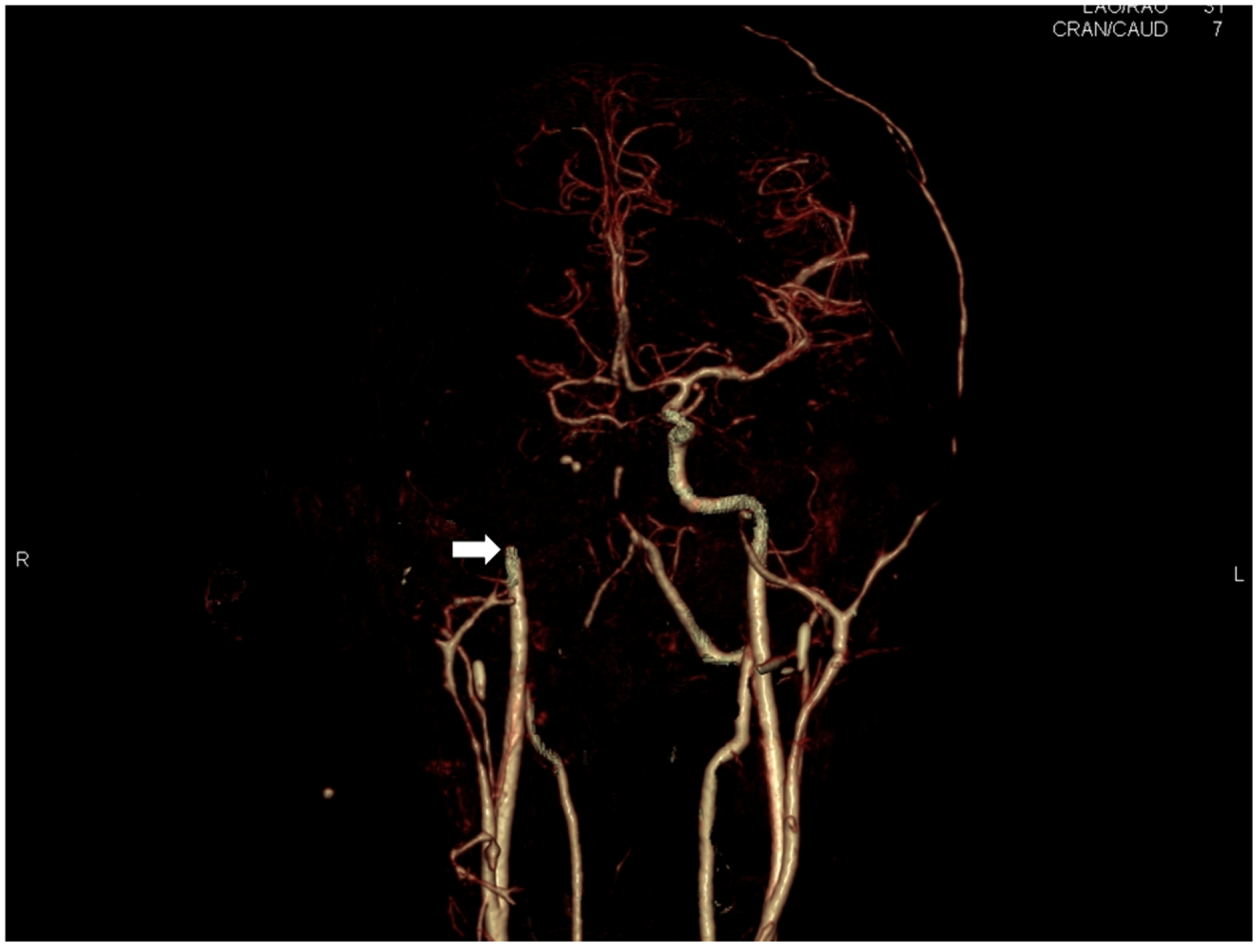
3D reconstruction of - cranial CT-Angiography after bone-removal. The white arrow shows the occlusion of the right internal carotid artery (white arrow) and the lack of vessel opacification in the right hemisphere.

## Discussion

**Figure 16:**
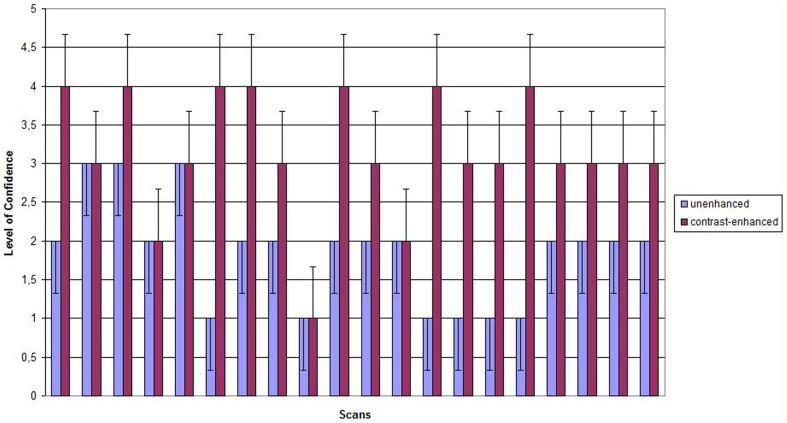
Comparison of LOC in unenhanced and contrast-enhanced scans. Level of confidence for each scan with error bars. The red bars indicate contrast-enhanced scans; the blue bars indicate non-enhanced scans, showing a significantly higher LOC for contrast enhanced scans (p = 0,001).

Postmortem intraarterial contrast enhancement and pulmonary ventilation have proven to be feasible and rather easy to manage. Intraarterial contrast enhancement significantly improves diagnostic accuracy of postmortem Computed Tomography in comparison to unenhanced scans as can be seen in the increase of LOC (Figure 16).

The correct diagnosis could be found with contrast enhanced postmortem computed tomography in all of the patients who died of cardiovascular pathologies. In two other cases, PMCT could not determine the cause of death.

In 7 out of 20 cases, the diagnosis could only be made with certainty due to contrast enhancement (35%). In 12 out of 20 Patients, contrast enhancement helped in the determination of the cause of death (60%). In the remaining case, contrast enhancement had no influence on the determination of the cause of death (5%).

The ventilation of the lungs helped to differentiate between post-mortem collapse of dorsal parts of the lung or antemortem ground glass opacification due to pulmonary edema (Figure 13a,b). Further, distension of the lungs helped in the visualization of pulmonary and mediastinal pathologies, as it allowed a much clearer differentiation of thoracal anatomy.

16 out of 20 Patients died due to cardiovascular pathologies (80%). 2 died due to cancer-associated multi organ failure (10%). One died of toxic shock syndrome (5%). In all 16 Patients who died of cardiovascular diseases, the cause of death could be found through postmortem contrast enhanced computed tomography. In both cancer patients, the tumours i.e. pulmonary metastases could be seen. In the patient who died of toxic shock syndrome, no definite cause of death could be diagnosed through postmortem computed tomography.

Some pathologic changes could be found through PMCT, that conventional autopsy did not find. Figures 9 and 10 depict air in the right ventricle and the sternal cavity due to suture insuffisciency of the sternotomy. This had not been found in conventional autopsy. As the application of contrast agent was performed using a closed system, and as there were no other air bubbles in the vascular system of neither this patient nor any other patients, an unwanted accidental injection of air in these quantities seems highly unlikely. Stenoses of iliac and carotid arteries remained unseen in conventional autopsy but could very well be seen in PMCT. Pulmonary ventilation did distend the thorax, establishing almost normal anatomic conditions, thus helping with the assessment of thoracic pathologies. In this study, the effect of pulmonary ventilation has not been statistically analyzed. This will be subject to further evaluation.

PMCT holds the possibility to assess tumour burden of entire organ systems such as bone metastases in prostate carcinoma or plasmocytoma, whereas in conventional autopsy, only samples are being evaluated. In PMCT all data remains stored to be viewed and assessed again, if necessary.

Other than with oily liquids, the contrast medium invented in this study showed no extravasation into the interstitial space [18] whereas it histologically proved to enter even smallest arteries such as the renal glomeruli. Unlike other water-soluble contrast agents, the newly invented contrast agent showed a very good perfusion of even smallest vessels but no extravasation and no shunting to the venous system [24]. thus being superior to other contrast agents described in the literature in regards to these effects.

When taking into account the benefit of pulmonary ventilation and contrast enhancement in regard to the cause of death, it becomes clear that both additions to the method help most in the diagnosis of cardiovascularly induced deaths. In the patients who died due to cardiovascular pathologies, contrast enhancement allowed for the correct diagnosis of 80% and helped in the diagnosis in the remaining three patients.

Lung metastases could very well be depicted due to ventilation and consecutive unfolding of the lungs. Pulmonary ventilation further provided a distended thorax, thus establishing almost normal anatomical circumstances. Contrast enhancement had no effect on the diagnosis of the cause of death in a patient who died of toxic shock syndrome.

Compared to each other, contrast enhanced scans provided more correct diagnoses than unenhanced scans. Further, the LOC improved significantly thus not only providing more correct diagnoses but a higher security in the determination of COD.

### Limitations

Due to subtotal obstruction of the femoral arteries because of sheaths in both sides and retrograde flow in the right femoral artery, the right leg did not show the same contrast enhancement as the left in 5 cases. Ruder et al. solve this problem by turning the sheath inside the vessel after body perfusion, what had not been done in this study [25].

Other authors favour the use of contrast pumps to administer contrast agent, which had not been done here. Nevertheless, the contrast agent did reach smallest vessels as described above. Venous thrombosis might have been missed as there was no venous contrast enhancement. There was no direct comparison between ventilated and non-ventilated lungs in this study. But as the previous study on this subject [10] patients did not receive pulmonary ventilation, the authors had substantial experience in both methods. Ventilation has not been formally assessed. This will be subject to further studies.

### Conclusion

Contrast enhanced postmortem computed tomography proved to be a very valuable method in the diagnosis of cardiovascular pathologies. It surpasses unenhanced PMCT in the Level of Confidence as well as the percentage of correct diagnoses.

Now, for the first time, the entire arterial vascular system can be visualized, seen, assessed and the data been stored for further evaluation. Thus, vascular pathologies such as occluded bypasses can be visualized which could not be done in unenhanced PMCT. What is more, different radiologists and pathologists can re-assess all data, thus evaluating the cause of death in a less observer-dependant way. As shown above, contrast enhanced PMCT with pulmonary ventilation displays its highest benefit in the diagnosis of cardiovascular pathologies, surpassing unenhanced scans in diagnostic accuracy and level of confidence PMCT already surpasses conventional autopsy in some fields, as it is a more comprehensive way to assess entire organ systems such as the skeletal system. It shows generalized bone pathologies entirely, whereas Conventional Autopsy can only show samples. But as conventional autopsy is still unsurpassed as a tool of quality control and in the determination of the cause of death, contrast enhanced PMCT is on its way to close the gap between virtual and conventional autopsy. Until now, both methods should be seen as complementary, each one adding valuable information to the other.
